# Influencing Holistic Health Policy

**DOI:** 10.1100/tsw.2007.205

**Published:** 2007-09-17

**Authors:** Erica Bell

**Affiliations:** University Department of Rural Health, University of Tasmania, Australia

**Keywords:** holistic health policy, research policy transfer, evidence into policy

## Abstract

Beliefs that health policy-making is an inherently ‘ideological’ or ‘irrational’ process appear to have worked to prevent researchers from developing better understandings of the kind of evidence that does work to influence policy. Without a model of policy-making that positions policy decision-makers as capable of being informed by specific forms of evidence that speak to policy contexts, it is difficult for research to begin to shape health policy. Recent years have seen the development of a research industry that focuses on developing and describing research approaches for shaping health and social services policy. This analysis paper offers a highly selective overview of generic features of policy-relevant research for holistic health. It aims to support efforts to develop better evidence for health policy by exploring elements of the genre of policy-relevant research, particularly as it applies to the challenges of holistic health policy-making. First, it offers a conceptual definition of holistic health policy-making, as well as research evidence for this kind of policy making, identifying some of the generic features of policy-relevant research. Second, it outlines some of the key practices for delivering sound evidence for health policy, in ways that highlight the salient differences between doing research for holistic health policy, and doing academic research in health. The paper concludes with directions for developing better evidence for holistic health policy-making that question the assumptions of quality which often inform elite funding agencies, calling for their diversification.

## INTRODUCTION

Evans and Stoddart conclude their watershed paper on the production of research for health policy with the view that

> ‘...There are other reasons why things do not happen, reasons that will not disappear in the light of advancing knowledge alone. Well-defined, though narrowly based, economic interests will be threatened by any serious efforts to act on the nonmedical determinants of health. Lavis et.al.[26] remind us of this reality, quoting the eminent American philosopher Homer Simpson: "Just because I don't care doesn't mean I don't understand."[1].

There is much ostensible truth in this statement, however such beliefs may have prevented researchers from better understanding the kind of evidence that does work to influence policy. Many health researchers are poorly equipped for the demands of doing research for policy, partly because they are trained in classical biomedical traditions that do not engage so well with the holistic concerns of health policy-makers, who speak the language of ‘continuum of care models’ and ‘inter-professional case management approaches’ and ‘whole-of-community frameworks’. There is sometimes a mistaken belief that training in academic research—bio-medical or any other academic discipline—necessarily imparts advanced-level skills in delivering policy-relevant research. Without a model of policy-making that positions policy decision-makers as capable of being informed by specific forms of evidence that speak to policy contexts, it is difficult for research to begin to shape health policy.

There are signs in the health policy literature that this view is gaining ground[2]. Recent years have seen the development of a research industry that focuses on developing and describing research approaches for shaping health and social services policy. A number of important monographs have been published since 2000, when an influential UK-based group associated with Sandra Nutley and Huw Davies published their edited collection *What Works? Evidence Based Policy and Practice in Public Services*[3]. These book-length studies have recognised the particular nature of both qualitative and quantitative research for policy-making contexts[3,4,5,6]. As well, across many sub-specialities of health, where holistic health challenges present complex methodological demands, ranging from as adolescent substance abuse to clinical error management and quality assurance in hospitals, there is increasing dissatisfaction with the social relevance of traditional research methods, and increasing recognition of a divide between research and policy and practice[7,8,9]. In great part, this relates to the increasingly apparent methodological limitations of both traditional qualitative and quantitative methods for studying context: the contexts of clients, their services, and their communities. In holistic health, these limitations are leading to explorations of new approaches, such as case-based qual-quantitative methods associated with USA-based sociologist Charles Ragin, which have only just begun to be applied to health[7,10,11,12]. Across other social science disciplines such as political science, history, and sociology, case-based methods, which aim to keep the configurational integrity of cases in small-*N* studies (including sub-group analyses within large-*N* studies), are gaining momentum as alternatives to traditional quantitative and qualitative approaches. These new approaches take a strong focus on theory-building using contingent generalisations useful to policy contexts[13].

The challenges of getting better research-policy translation are not simply about culture and methodological divides between academic and policy research contexts. They also relate to getting better in-practice and conceptual understandings of the emerging genre of policy-relevant research often produced by public sector research units whose existence depends on their capacity to deliver evidence that has utility for policy-makers. This genre is poorly theorised even, and especially, within the public sector research cultures where it has flourished. This paper is designed to make a contribution to this last challenge by exploring elements of the genre, particularly as it applies to the challenges of holistic health policy-making. That is, this paper aims to explore some aspects of the craft of delivering research evidence for policy-making. First, it offers a conceptual definition of holistic health policy-making, as well as research evidence for this kind of policy-making, identifying some of the generic features of policy-relevant research. Second, it outlines some of the key practices for delivering sound evidence for health policy, in ways that highlight the salient differences between doing research for holistic health policy, and doing academic research in health. The paper concludes with directions for developing better evidence for holistic health policy-making.

## METHODS

This analysis paper does not offer a review; rather, it aims to give a highly selective overview of generic features of policy-relevant research for holistic health. As such, its content has been developed inductively via a number of pathways:

- building on the author's work in previous papers on methodological debates in different sub-specialities of health, as cited in this paper, as well as her applied policy development background in health and education as a consultant and public sector policy research manager

- scanning of abstracts and papers from peer-reviewed journals using PUBMED database searches on various terms: 'health policy research methods' (3,030 references), 'holistic health policy' (146 references), 'research utilisation policy'(124 references) and 'research policy transfer' (714 references): these scans suggested that there has been very little published giving an overview of the generic features of research designed to meet the needs of for holistic health policy-making; in contrast, a great deal has been written about specific content areas of health policy-making, particularly the experiences of different countries and systems developing policy in particular areas; this exercise helped clarify the contribution such a paper could make and its rationale

- monograph searches of Amazon and Barnes and Noble websites using the term 'health policy' for the period 2000-2007

- Google Scholar searches on the terms 'Alexander George' and 'Charles Ragin' to gain an overview of publications by both these case-based theorists, whose work features in policy and theory building debates; a selection of references from the body of writing by Charles Ragin's work was made using the list available on his website at http://www.u.arizona.edu/~cragin/cragin/

## RESULTS

### Conceptual Understandings of Holistic Health Policy-Making

Holistic health policy-making is about an engagement with the bio-medical, as well as the non bio-medical, nature of holistic health challenges. If holistic health practice is practice informed by the bio-psycho-social determinants of health as part of a 'whole-of-patient' approach, holistic health policy-making is about an engagement with client-service-community determinants of health as part of a 'whole-of-systems' approach[14].

Holistic health policy-making cannot be rightly understood as a single amorphous entity. Rather, it operates at different levels and is characterised by different styles of policy-making, in different organisational settings and forums. However, health policy-making that could be described as 'holistic' shares an engagement with client-service-community interactions in responding to health challenges that have complex multidisciplinary causality. Accordingly, holistic health policy-making is also characterised by a conceptualisation of health challenges as requiring 'whole-of-systems' approaches that mobilise not simply single specialist clinical units to meet health challenges, but rather multiple health and allied social services that can deliver multi-disciplinary responses tailored to meet the individual client's health needs.

It is sometimes mistakenly supposed that policy-makers operate in 'rational' ways, when they use research evidence and 'irrational' ways when they do not. In the policy research literature, this popular distinction has been most powerfully dismantled by Ian Hunter and his colleagues at Griffith University's Institute for Cultural Policy Studies in their monograph exploring policy changes in Australian higher education in the late 20th Century[15]. Hunter used the theorisations of French philosopher Foucault as a point of departure for arguing that higher education policy-making in Australia has been formed by different styles of rationality expressed in the techniques of power characterising the period. In the light of Hunter's work, it appears that the characterisation of policy-making as too frequently 'irrational' or 'ideological' in nature has prevented researchers from engaging with the specific styles of rationality that variously characterise governance, service, and practice levels of policy-making, in the different settings of government, not-for-profit, and private enterprise sectors, and in local, state, national, and international forums. Hunter's work suggests that health research that does not engage with the specific rationalities or the 'logic' of policy-makers has poor transferability for the contexts of policy.

The policy literature suggests that different academic research genres have specific problems of transferability to policy-making contexts[16]. Studies of the research evidence health policy-makers use suggest they are informed by different kinds of evidence[17]. This includes the evidence from investigations of grievances raised by health consumers; the results of systematic literature reviews in academic journals; clinical practice guidelines; as well as state and national reviews and investigations commissioned by policy-makers from experienced policy researchers. This last-mentioned genre is growing in sophistication yet lacks an authoritative body of theory and evidence about how it works. The genre could be described as taking a solutions-driven focus on policy challenges relevant to a specific context. Sometimes this context is local, as in the development of health and allied health services for child health in a specific community in relation to a specific social challenge such as domestic violence[18]; sometimes the focus is international, as in the work of the United Nations developing policies and guidelines for substance abuse service development[19,20]. The centrality of a specific policy problem in such research evidence defines its many other features, such as its preoccupation with weighing the value of alternative models, or the findings of community consultations on the possible policy options under consideration. Throughout, such research evidence displays an engagement with the specific 'logic' defining health policy-making in that context: the weighted configuration of health, financial, political, social, historical and other factors important to the particular policy-makers to whom the evidence is being delivered.

## Practical aspects of delivering evidence for holistic health policy-making

A critical first step in delivering evidence for holistic health policy-making is deciphering the policy challenge or problem. This is equivalent to the requirement for a clearly defined research question in academic genres. Without an understanding of the policy challenge the researcher cannot design a research method or write a report that speaks to the policy challenge under consideration by policy-makers. Understanding the policy challenge is about understanding the local context; a lack of appreciation of the structures and constraints under which policy makers work is a key reason for research being unusable by policy-makers[21-26]. In policy contexts, the policy challenge must be deciphered from written and unwritten texts. Such texts will relate to clients, services, and communities, and will require multi-disciplinary skills from the researcher. The researcher needs to understand the production of the policy challenge not just in bio-medical terms, but also in available economic, sociological, historical, and political science texts, overt and covert, in order to decipher the holistic policy challenge. For example, if the researcher is asked to review a state health system with a view to developing better mental health services for senior citizens, the critical questions for interrogating these diverse texts will most likely include:

- what is best practice for geriatric mental health as described in the clinical bio-medical literature?
- what is best practice for geriatric mental health in the wider health and allied health literature, including the social services literature?

- what do the different community stakeholder groups, such as practitioners and consumer advocacy groups around the state, believe about best practice in geriatric mental health?

- what historical experiences have shaped community beliefs and services in this area, for example, previous experiences with different models of service delivery?

- what relevant extant models of service delivery exist in the local community and around the world? What is the available evidence for their clinical, economic, social, and political effectiveness?

- what are the political and financial constraints on health policy-makers that need to be considered?

Such questions will not all answerable at the outset of the research: rather, the answers will emerge as part of the iterative process that is common to all research. In holistic health policy-making contexts, however, the sources to which researchers must pay attention are substantively different, as are the multidisciplinary skills required to read these sources accurately.

Accordingly, the 'literature review' used in health policy-making research will involve an attention to both scholarly as well and non-scholarly sources from multiple disciplines. For example, many policy-critical features of working health services of interest in developing health policy options are poorly documented in the research literature. This means that the researcher will need to excavate available information about exemplar health services through direct contact with agencies across the globe. In such a review, the quality of the research in which possible alternative models are described matters less than the substantive relevance of the actual model of practice, and the accuracy of practice details about it, for solving the policy problem under consideration.

The design of research methods for holistic health policy-making involves particular challenges. Classical statistical techniques deliver variable-driven analyses that offer limited 'big picture' information for macro-policy formation. This is because they require large datasets for few variables to have the necessary statistical 'power'. The problems with such methods for nuanced policy-making contexts is that they fail to offer the configurational information that policy-makers require i.e. they fail to answer the question 'Which elements of different health systems models can be combined to produce a system that resolves, even partly, the policy problem in this community?' Answering this kind of question involves using research methods that are capable of capturing client-service-community interactions—not a hallmark of the methods characterising clinical trials which are highly valued by many health funding agencies and dominate the health sciences.

In contrast, qualitative approaches have value for capturing contextual complexity, because they allow for development of detailed knowledge about single or small numbers of cases through observational and interpretative methods. However, policy-making is by definition 'high stakes' decision-making. This means that qualitative methods lack the reliability and generalisability that may be critical to such decision-making.

One common solution is to use both qualitative and quantitative methods, designing 'mixed methods' approaches that include crosschecks of policy-critical hypotheses. However, this by itself may not be enough to overcome the limitations of both traditions for holistic policy-making. If researchers for holistic health policy cannot afford to refine a single methodological tool throughout their careers, they also cannot afford to rest on tradition and mono-disciplinary knowledge. They must range far beyond the discipline of health—wedded to beliefs that clinical trials are the gold standard—and engage with debates about methodology emerging in the political sciences, history, and sociology. This paper has suggested that the most significant of these debates are now occurring around the development of case-based analysis: most notably in the writings of Charles Ragin detailing his Qualitative Comparative Analysis (QCA) techniques which focus on summarising the configurational complexity of cases[10,27,28,29,30], as well as in the writings of Alexander George and Andrew Bennett exploring 'process tracing' as part of an archaeological or historical approach to the study of particular cases[13]. The potential of such approaches for holistic health policy is largely untapped. However, they have great potential value for critical tasks in policy-relevant research such as

- configurational analysis of the available alternative models of health in ways that keep their integrity as cases yet allow for rigorous comparisons that can inform policy reviews

- the development of typologies of health service systems and models, in conceptualisations that help generate understandings of possibilities for policy-making

- summarising relationships between the attributes of health systems/models and the outcomes they are known to achieve for their communities, in ways that help develop preferred policy options.

Two key elements of designing research evidence for policy-making are ‘consensus-finding’ and ‘consensus building’. ‘Consensus-finding’ is investigative and diagnostic research: it is about finding out what are the community views around a particular set of policy options. However, ‘consensus-making’ is about creating community consensus through consultative research techniques. Commonly, the Delphi technique has been used in health to obtain practitioner or expert consensus on complex judgments not resolvable with more direct evidence[31]. However, for community consultations, researchers will need an advanced command of other qualitative methods useful to textual exegesis such as ethnography, hermeneutics, and discourse analysis. Not only that, they will also need to be able to apply these methods in ways that are complementary to quantitative and case-based approaches. For example, the results of consensus finding and consensus-making involving key community stakeholders need to speak to the findings about the viability of alternative service models in other systems. This is because research evidence for policy needs to build a policy argument using complementary data analyses.

The production of findings from multiple approaches—qualitative, quantitative, and case-based—is another critical element of research for policy-making. The full range of evidence, including community views and policy possibilities need to be packaged in a ‘policy story’ that is conversant of community and media sensitivities. The findings need to be ordered hierarchically: some are macro-level findings about broad system reforms, while others are micro-level findings about key aspects of implementation of possible policy options. The findings need to be organised so they persuasively convey the policy argument packaged as a ‘policy story’ i.e. present the preferred policy options and evidence for them while canvassing the broad range of policy challenges and the different kinds of evidence for their solutions.

Policy options represent a distinctive aspect of the genre of policy-relevant research. Ideally, they will take a combinatorial form: each policy option can model a possible policy intervention designed by combining different features of health systems and models explored in the body of the report, for which there is likely to be community support. The policy options should be presented with a snapshot of the arguments for and against each option. This snapshot should not contain any material not already well developed in the body of the report. However, there will be limits to the ‘evidence base’ available for new policy options. Policy decision-making is a kind of theory-bound forecasting in the sense that it most often involves an integrated set of assumptions about how a newly designed model will work in conditions where it has not operated before. This feature of policy decision-making—the need to make a reasonable ‘bet’ that a new combination of different elements will work in a new context or community—sharply distinguishes it from other forms of judgments made in other health contexts, such as clinical practitioner judgments to administer medications that are supported by evidence from randomised controlled trials involving patients with ostensibly the same condition. The nature of the judgment involved in holistic health decision-making makes the delivery of evidence both in the body of the report and the summaries supporting the policy options particularly demanding. This does not mean that holistic health policy-making is so ‘holistic’ as to lack a systematic reasoning pathway. For example, consideration of policy reviews demonstrates that they place a high value on inductive reasoning pathways in evidence weighing, triangulating, and integrating the findings of diverse complementary methods[32]. The critical task for researchers then is to model these inductive reasoning pathways in the delivery of the evidence, through the adroit structuring of the policy argument throughout the report such that they maximise the extent to which the policy is 'evidence-informed' or at least 'evidence-aware'[33]. This inductive development of evidence for the policy options will be critical, for policy-makers will be asked by the media and the community what 'evidence' there is that the proposed new model will work for that community.

## CONCLUSIONS

This paper has emphasised the particular nature of policy-relevant research. In cultures where policy-making is under pressure to be (at least ostensibly) evidence-based, the distinction between high quality policy-relevant research and research that has poor utility for policy-making is an important one. The traditional background of health researchers presents particular challenges for developing better evidence for holistic health policy challenges. What practical steps can be taken to develop better holistic health policy?

Clearly, a key direction is for primary health care training and professional development courses to include an emphasis on the acquisition of policy-relevant research skills taught by experienced applied policy researchers. The recruitment of trainers for such courses should not assume that high-level researchers at professorial level necessarily have the knowledge and skills in teaching policy-relevant research. Creating the conditions where such applied policy researchers are recognised as having something to offer health research training will involve a cultural shift. Yet, as this paper has argued, there are signs that the assumption that skills in clinical research necessarily imply skills in policy-relevant research is being questioned.

Another key direction relates to elite funding agencies developing evidence-based assessments of the merit of applied policy research publications presently 'not counted' because they fall outside definitions of academic research quality (peer-reviewed journal papers and scholarly monographs). The notion that socially relevant research is going to be delivered by research funding systems that do not classify much socially relevant research as quality research is a problematic one for getting better holistic health policy. Too often, these assumptions are based on anecdote and vaguely articulated prejudices, rather than systematic reviews comparing the quality of, for example, applied policy research published by public sector agencies (often after extensive review by multiple committees comprised of diverse experts) with that produced for academic journals (often after review by three or four experts at most). Such evidence-based analyses of the quality of applied policy research published outside academic domains should be cognisant of the specific generic constraints under which this research is applied. Recognising these constraints is not about lowering standards, but rather making room for other kinds of gold standards in order to ensure that research better serves its communities.

Another direction is for elite funding agencies to prioritise the funding of projects that develop research methods for policy, particularly case-based approaches used to extend qualitative and quantitative methods. This is happening in some countries, to some extent, however, many researchers trying to develop innovative methods that break free of conservative health research cultures still face difficulties because they are not reproducing a singular gold standard for health research—classical, big-*N* experimental studies using traditional quantitative techniques. Dedicated funding for research programs that develop better methods for health policy contexts, with suitably diverse review panels and criteria that recognise policy-relevant research would be a good first step.

In short, what is required for better evidence for the styles of holistic health policy-making now dominating policy landscapes is a rethinking and diversification of the notions of quality that inform academic cultures and funding systems. Without this, it seems unlikely that health policy, and therefore the health systems our communities experience, will be informed by the best possible research evidence.
